# Substrate specificity of plant nitrilase complexes is affected by their helical twist

**DOI:** 10.1038/s42003-018-0186-4

**Published:** 2018-11-02

**Authors:** Jeremy D. Woodward, Inga Trompetter, B. Trevor Sewell, Markus Piotrowski

**Affiliations:** 1 Division of Medical Biochemistry and Structural Biology, Department of Integrative Biomedical Sciences, University of Cape Town, Anzio Road, Observatory, Cape Town, 7925 South Africa; 2 Department of Molecular Genetics and Physiology of Plants, Ruhr-Universität Bochum, Universitätsstr. 150, 44801 Bochum, Germany; 3 Institute of Infectious Disease and Molecular Medicine, University of Cape Town, Anzio Road, Observatory, Cape Town, 7925 South Africa

## Abstract

Nitrilases are oligomeric, helix-forming enzymes from plants, fungi and bacteria that are involved in the metabolism of various natural and artificial nitriles. These biotechnologically important enzymes are often specific for certain substrates, but directed attempts at modifying their substrate specificities by exchanging binding pocket residues have been largely unsuccessful. Thus, the basis for their selectivity is still unknown. Here we show, based on work with two highly similar nitrilases from the plant *Capsella rubella*, that modifying nitrilase helical twist, either by exchanging an interface residue or by imposing a different twist, without altering any binding pocket residues, changes substrate preference. We reveal that helical twist and substrate size correlate and when binding pocket residues are exchanged between two nitrilases that show the same twist but different specificities, their specificities change. Based on these findings we propose that helical twist influences the overall size of the binding pocket.

## Abstract (web summary)

Jeremy Woodward et al. report that substrate specificity in nitrilases from *Capsella rubella* depend on their helical twist rather than substitutions in the substrate-binding site. They show that mutating interfacial residues can alter substrate specificity and helical twist.

## Introduction

**Fig. 1:**
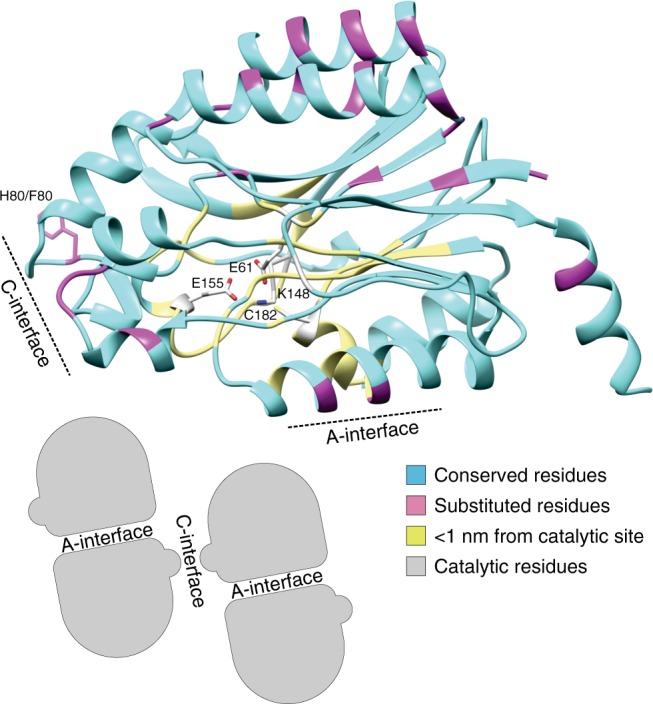
Structural model of a plant NIT1-group nitrilase. E61, K148, E155, and C182 form the known catalytic tetrad^46^. Positions in magenta indicate residue differences between *Cr*NIT1 and *Cr*NIT2 while cyan indicates identical residues. Positions in yellow are identical between the two enzymes and lie within a radius of 1 nm of the catalytic cysteine (C182). The inset shows the topology of the interfaces of the nitrilase monomers in the supramolecular spirals or helices

Substrate specificity is one of the hallmarks of enzymes, the catalysts of the living cell. Understanding the principles of substrate recognition is an important step towards understanding how enzymes have been neo-functionalized during evolution and is necessary for directed attempts at changing substrate specificity, e.g., for biotechnological applications in biocatalysis. We are interested in the factors that define substrate specificity in nitrilases (EC 3.5.5.1), members of the C–N hydrolase superfamily^1,2^. Nitrilases are nitrile-hydrolyzing enzymes (producing carboxylic acids and/or their corresponding amides), which seem to be ubiquitous in plants and fungi and are often found in bacteria^3–5^. In vitro, nitrilases (and the closely related cyanide [di]hydratases) from bacteria and fungi form either short spirals consisting of 8 to 22 subunits or long filaments^6–13^. The basic unit of these supramolecular complexes consists of dimers in which the monomers associate across an interface that is preserved in most members of the superfamily (A-interface, Fig. 1). Oligomerization of the dimers leading to the formation of spirals or helical fibers seems to be an important prerequisite for nitrilase activity^7,14,15^. Interestingly, nitrilases from plants of the *Poceaea* (grass) family must form heterocomplexes to be active^16^ indicating that heterologous, interfacial interactions between the monomers influence the active site.

Nitrilases are enzymes of biotechnological interest because of their ability to convert easily synthesized nitriles to high-value organic amides and acids that are used industrially as drug precursors or fine chemicals^5,17,18^. While nitrilase catalytic activity, stability, by-product formation, and enantiospecificity have been altered to render enzymes more amenable to industrial processes (e.g., Kiziak and Stoltz^19^), attempts to alter substrate specificity have so far met with limited success^20–22^. Novel substrate specificities are therefore still generally found by screening bacterial and/or fungal nitrilases from environmental samples^23^. However, the substrates obtained are unlikely to be the natural substrates for these enzymes. In fact, the natural substrates of bacterial and fungal nitrilases are largely unknown and little has been learned about the basic principles of nitrilase substrate specificity from these experiments.

In contrast, the natural substrates for many plant nitrilases have been identified and the substrate specificities of the respective enzymes have been well characterized. The typical plant NITRILASE 4 (NIT4) enzymes have high specificity for their substrate β-cyanoalanine, which is an intermediate product in the plant’s cyanide detoxification pathway^24,25^. Other plant nitrilases have evolved to hydrolyze substrates that are derived from secondary metabolites^16,26,27^. One example of such nitrilases are the NITRILASE 1 (NIT1) group enzymes, which were identified for the first time in *Arabidopsis thaliana*, where they form a small family consisting of three members (*At*NIT1, *At*NIT2, *At*NIT3)^28,29^. NIT1-group enzymes, which are restricted to plants of the *Brassicaceae* family^26^, are possibly involved in the catabolism of nitriles derived from glucosinolates^4,26,27^, which are the typical secondary metabolites in these plants. More than 120 different glucosinolates have been described thus far, making NIT1-group enzymes an interesting subject for studying enzyme evolution. In addition, sequence identity of plant nitrilases is very high (ranging from 54% to 98%—usually above 70%), which allows for the identification of signature sequences for enzymes with different substrate specificities. Thus, plant nitrilases are a good model system to study the basis for substrate specificity in the enzyme class. Despite their high sequence similarity, our previous attempts to change substrate specificity in plant nitrilases by exchanging residues within the proposed substrate-binding pocket proved unsuccessful, hinting that there may be some other factor(s) affecting substrate-binding-site architecture.

Here, we report our work on two highly homologous nitrilases with distinct substrate specificities from the plant *Capsella rubella*. Our results demonstrate that an interface residue distant from the proposed substrate-binding site changes the helical twist of the supramolecular nitrilase complex, and affects substrate specificity. We present evidence that the helical twist determines the size of the substrate-binding site, and that even when the amino acid sequence of the enzyme remains unchanged, changing the twist can alter substrate specificity.

## Results

### One residue determines specificity in two plant nitrilases

**Fig. 2:**
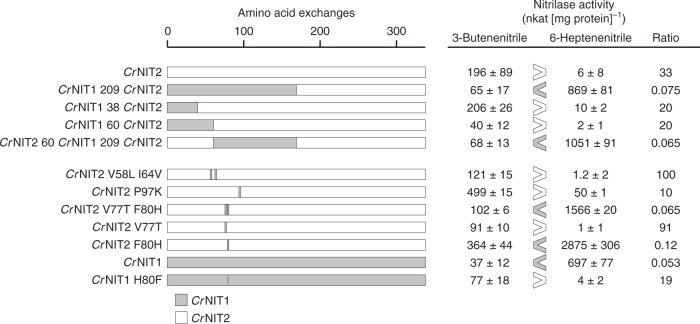
Screening for substrate-specifying residues. Gray (*Cr*NIT1) and white (*Cr*NIT2) indicate how the chimeras were constructed. Each mutant was tested for specific activity against 3-butenenitrile and 6-heptenenitrile. The activities are given in nkat (mg protein)^−1^ (mean ± sd). Preference for 3-butenenitrile or 6-heptenenitrile is indicated by > or <. A single amino acid residue, H80 in *Cr*NIT1 and F80 in *Cr*NIT2, appears to be responsible for determining the preferred substrate

We recently identified and characterized two nitrilases from Pink (or Red) Shepherd’s Purse (*Capsella rubella*) with 88% sequence identity, but different substrate specificities (Figs. 1, 2, and Supplementary Figure 1). *Capsella rubella* NITRILASE 1 (*Cr*NIT1) (XP_006291436.1) shows a preference for several aliphatic alkenenitriles with peak activity against 6-heptenenitrile, while *Cr*NIT2 (XP_006284056.1), also a member of the NIT1 group, displays a relatively high degree of specificity for 3-butenenitrile (Fig. 2 and Supplementary Table 1). The sequences of both enzymes have only 51 amino acid differences over their length of 342 residues of which 14 occur in the first 20 positions (Supplementary Figure 1). In order to narrow down which of these residue differences might account for the observed substrate preferences of the two enzymes, we decided to identify which of them occur within or close to the substrate-binding pocket.

We constructed a model of the *Cr*NIT monomer using the crystal structure of Nit6803 from *Synechocystis* sp. PCC6803^21^ (BAA10717.1, pdb id: 3wuy) as template (Fig. 1). Nit6803 is the only spiral/helix forming nitrilase enzyme for which a crystal structure is currently available, in addition it shares reasonable homology to the *Cr*NITs (35% identity). Amino acid residues potentially lining the active site pocket were identified by homology modeling on the basis of nitrilase superfamily members with bound substrates/intermediates: the C171A/V236A mutant of *N*-carbamyl-d-amino acid amidohydrolase with bound *N*-carbamyl-d-methionine (pdb id: 1uf5)^30^; the amidase from *Pseudomonas aeruginosa* with trapped acyl-transfer reaction intermediate (pdb id: 2uxy)^31^ and a C165A mutant of the amidase from *Nesterenkonia* AN1 with butyramide bound in the active site pocket (pdb id: 4izs)^32^ (Supplementary Figures 2 and 3). Strikingly, these results strongly indicated that there were no amino acid differences between *Cr*NIT1 and *Cr*NIT2 as far as 1 nm from the active-site pocket (Fig. 1 and Supplementary Figure 2).

In order to identify which amino acid exchanges were responsible for specifying substrate-specificity, we generated chimeras between the two enzymes by in vitro mutagenesis and screened for activity with 3-butenenitrile and 6-heptenenitrile. Using the binary search technique, a single amino acid residue in position 80 (H80 in *Cr*NIT1 and F80 in *Cr*NIT2) was identified, that when exchanged, led to an almost complete switch in substrate preference ratio (Fig. 2).

### Position 80 exerts an influence on the helical twist

Sequence and structural alignments (Supplementary Figures 1 and 2) and homology modeling allowed us to locate residue 80 at the interface between two nitrilase dimers (C-interface, Fig. 1). The precise nature of the interaction is unclear because plant nitrilases have an insertion of five amino acids relative to Nit6803 in the vicinity of residue 80 (Supplementary Figure 1). Nevertheless, this raises an important question about the basis for selectivity: why does an interface residue have such a marked effect on substrate specificity? In the absence of amino-acid changes within (or close to) the binding pocket, the change in the substrate specificity may result from a change in the three-dimensional location and/or orientation of the active site residues. We reasoned that this altered conformation might explain the observed change in affinity for longer or shorter substrates.

**Fig. 3:**
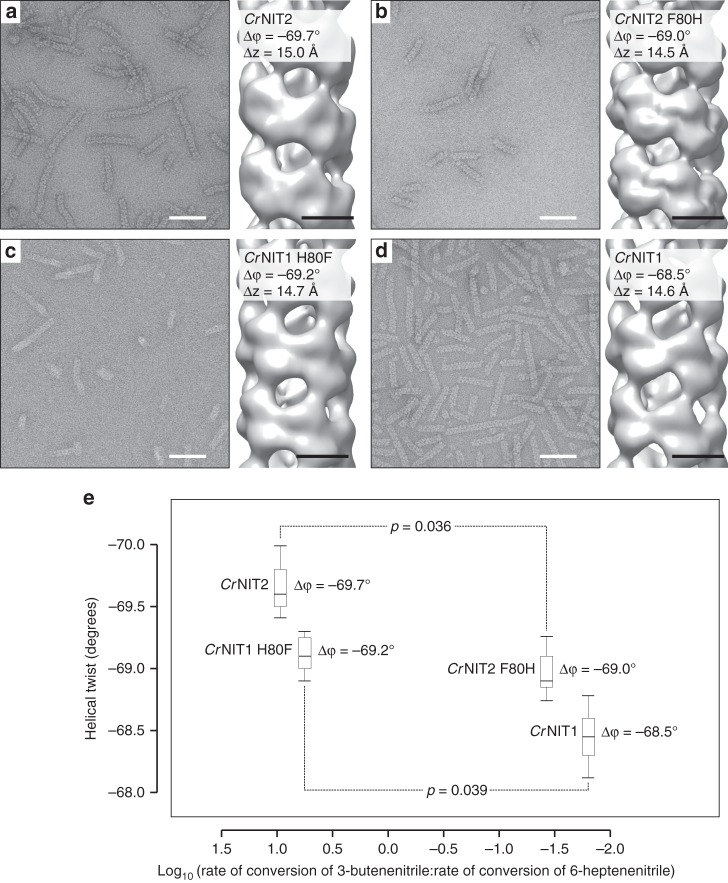
Electron micrographs of negatively-stained nitrilase fibers and three-dimensional reconstruction of the fiber structures. **a** *Cr*NIT2 (EMD-3501), **b** *Cr*NIT2 F80H (EMD-3503), **c** *Cr*NIT1 H80F (EMD-3500), **d** *Cr*NIT1 (EMD-3499). As with nitrilase enzymes in general, the fibers show a high degree of heterogeneity with respect to length, the *Cr*NIT1 fibers are better ordered (straighter) than the *Cr*NIT2 fibers, which as a result show a slightly elongated Δ*z*. The filaments consist of dimers arranged in a left-handed^9^, one-start helix. The angle of rotation between successive dimers about the helical axis defines the helical twist, Δφ, which, by convention, is negative in order to indicate left-handedness. Scale bars represent 50 nm on the electron micrographs, 5 nm on the reconstructed fibers. **e** The wild-type enzymes show a statistically significant (Student’s two-tailed unpaired *t*-test: *p* < 0.05) change in helical twist compared to the corresponding mutants (1st quartile, median and 2nd quartile ± sd). The two enzymes with larger twists (absolute values) show a preference for the shorter substrate and vice versa

To test this idea, we imaged *Cr*NIT1 and *Cr*NIT2 as well as *Cr*NIT1 H80F and *Cr*NIT2 F80H, in three dimensions by negative-stain electron microscopy (Fig. 3a–d). The wild-type enzymes were found to form long fibers with slightly different helical twists (Δφ) with |*Cr*NIT2| > |*Cr*NIT1| (absolute values) while the twist of the helices formed by the mutant enzymes were exchanged with |*Cr*NIT2 F80H| < |*Cr*NIT1 H80F| suggesting a relationship between specificity and helical arrangement (Fig. 3e and Supplementary Movie 1).

### Correlation of helical twist and substrate size

**Fig. 4:**
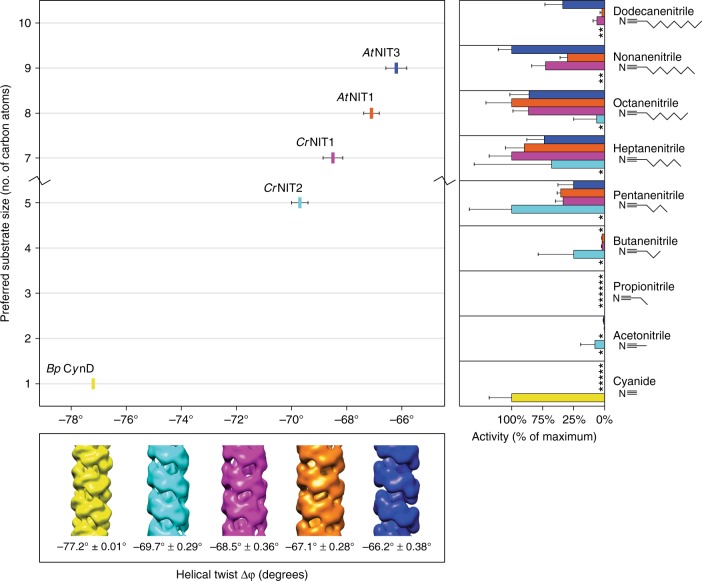
The relationship between helical twist and substrate length for a sample of nitrilases. Enzymes that favor larger substrates (mean ± sd) have smaller absolute helical twists (mean ± sd). The twists are given as negative numbers in order to indicate that the one-start helix of the fiber is left handed. (*) Indicates no detectible ammonia formation after 30 min incubation

Following this discovery we reconstructed additional helix-forming plant nitrilases with well-defined substrate specificities in three dimensions. For a better comparison we re-determined the substrate specificity of these enzymes using eight aliphatic saturated nitriles ranging from acetonitrile to dodecanenitrile (Fig. 4 and Supplementary Table 1). Of these, *Arabidopsis thaliana* NIT3 (NP_190018.1, EMD-3496), another member of the NIT1 group, has a generally low activity against a wide range of different substrates, but has a tendency to prefer larger substrates. *Arabidopsis thaliana* NIT1 (NP_851011.1, EMD-3486) has peak activity against the aliphatic substrate octanenitrile and the aromatic substrate 3-phenylpropionitrile^27^. We also included the known helical twist of the cyanide dihydratase *Bp* CynD from *Bacillus pumilus* (AAN77004.1), which is active against the smallest substrate (cyanide) and shows the tightest absolute value of helical twist (Δφ = −77.0°)^33^. In order of decreasing looseness, the helix opens up and the diameter of the fiber increases (Fig. 4). A clear correlation is observed between Δφ and the length of the R-group of the preferred substrate. Furthermore, it was observed that the greater the absolute value of the twist of the enzyme (and smaller the substrate) the more specific the enzyme tends to be. On the two extremes, *Bp* CynD is highly specific for cyanide while *At*NIT3 has broad substrate specificity. This observation can be extended to the NIT4 homologs, which are very specific for β-cyanoalanine (a rather short substrate) and likewise have a large absolute value of the twist (Δφ around −74°) (Supplementary Figure 4a-c).

### Imprinting a different twist changes substrate specificity

To further investigate the influence of the helical twist on substrate specificity, we performed mixing experiments with the *Capsella* nitrilases where one partner was in excess (in a ratio of 5:1) over the other. The rationale behind this is that the minority partner may become incorporated into helices of the majority partner and thus may be imprinted with the helical twist of the latter. To avoid the influence of the excess partner’s own enzymatic activity, we used inactive mutants in which the active-site cysteine was mutated to an alanine. Helical twist measurements on the inactive (C182A) mutants were not performed as we have previously observed in other nitrilases that helical twist was unaffected by these mutations.

**Fig. 5:**
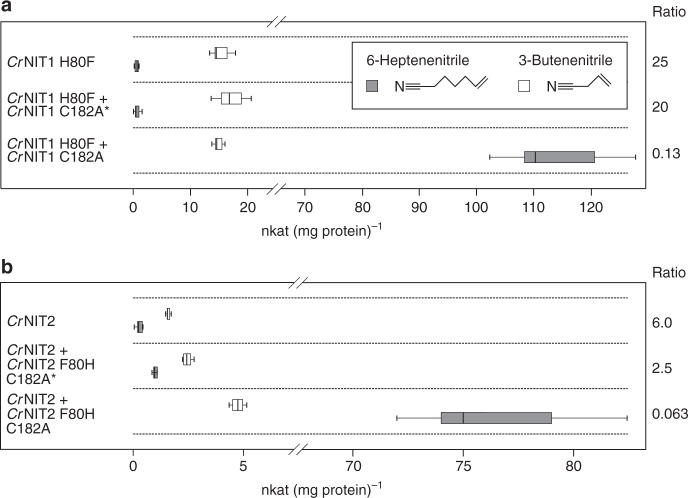
Imprinting a different twist changes substrate specificity. *Cr*NIT1 H80F (**a**) and *Cr*NIT2 (**b**) both have a preference for 3-butenenitrile. They were mixed with an excess of inactivated mutants (C182A) of enzymes that prefer 6-heptenenitrile, *Cr*NIT1 or *Cr*NIT2 F80H, respectively. In both cases, the active enzyme changes its substrate preference towards 6-heptenenitrile (1st quartile, median and 2nd quartile ± sd). Mixing with boiled enzyme (*) showed no effect. This experiment demonstrates that the minority component can assume the twist of the majority component and that its specificity is thereby altered

Indeed, when we mixed active *Cr*NIT1 H80F with an excess of an inactive mutant of *Cr*NIT1 (*Cr*NIT1 C182A) we found that the inactive enzyme transfers its substrate specificity to the active enzyme (Fig. 5a). The same result was obtained when mixing *Cr*NIT2 with inactive *Cr*NIT2 F80H (*Cr*NIT2 F80H C182A) (Fig. 5b). The generally low activities that were obtained in these experiments are the result of the relatively long enzyme pre-incubation times (in the absence of substrates) of 2 h at room temperature (*Cr*NIT1 H80F + *Cr*NIT1 C182A) or 37 °C (*Cr*NIT2 + *Cr*NIT2 F80H C182A). This pre-incubation of the mixtures is necessary to allow for the exchange of nitrilase monomers between the helices, but is obviously detrimental to enzyme activity.

### Changing specificity in nitrilases with similar twist

Our experiments demonstrate that the role of helical twist in defining substrate specificity has some generality for the subset of nitrilases tested. While the twist appears to define the overall size of the active-site pocket, the details of the interactions that influence binding are likely to be specified by the residues lining the substrate-binding pocket. We have identified another plant nitrilase, *Sinapis alba* NITRILASE 1–3 (*Sal*NIT1–3) (MH567030) (Supplementary Figure 1), with high activity against 4-hydroxyphenylacetonitrile (see below), and which has a broadly similar helical twist (Δφ = −70.6°) to *Cr*NIT2 (Supplementary Figure 4d) (EMD-3505). Thus, the twist of both enzymes differs by ~0.7° but they show a completely different substrate preference.

**Fig. 6:**
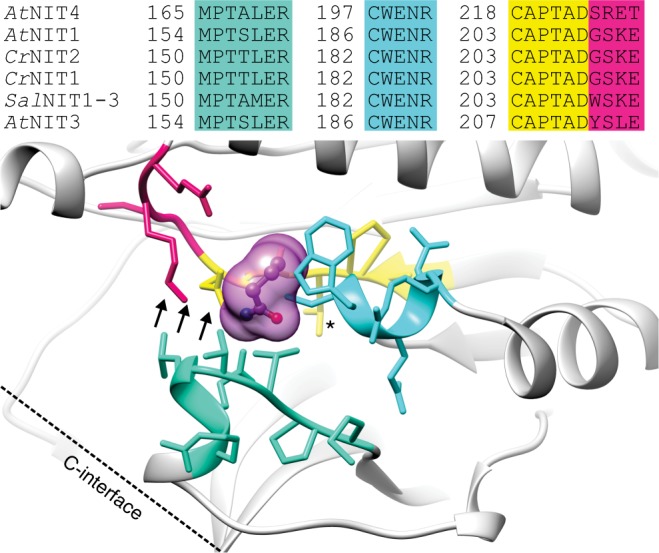
Nitrilase regions in close proximity to the bound substrate. Homology model of *Cr*NIT2 with superimposed butyramide from the active site of the C145A mutant of the amidase from *Nesterenkonia* AN1 (pdb id: 4izs). Regions that interact directly with the substrate are colored turquoise (α4), cyan (α5), yellow (loop after β8), and magenta (loop before α6). Increasing the helical twist most likely results in the movement illustrated by the arrows. Two of the four regions identified vary between different plant nitrilases (inset)

Homology modeling of the nitrilases under investigation on the basis of nitrilase superfamily members with bound substrates/intermediates (Supplementary Figures 2 and 3) identified four regions that line the substrate-binding pocket (Fig. 6). Two amino acid motifs differ between *Cr*NIT2 and *Sal*NIT1-3, the first shown in turquoise defines the outer edge of the substrate-binding pocket. In the case of *Cr*NIT2, this motif has the sequence MPTTLER while the corresponding motif in *Sal*NIT1-3 has the sequence MPTAMER. The second active site motif shown in magenta differs by a single amino acid substitution, reading: GSKE (*Cr*NIT2) or WSKE (*Sal*NIT1-3). We exchanged these motifs by site-directed mutagenesis between *Cr*NIT2 and *Sal*NIT1-3 and assessed the effect of each mutation on substrate specificity by measuring the specific activity of the mutants against 4-hydroxyphenolacetonitrile and 3-butenenitrile.

**Fig. 7:**
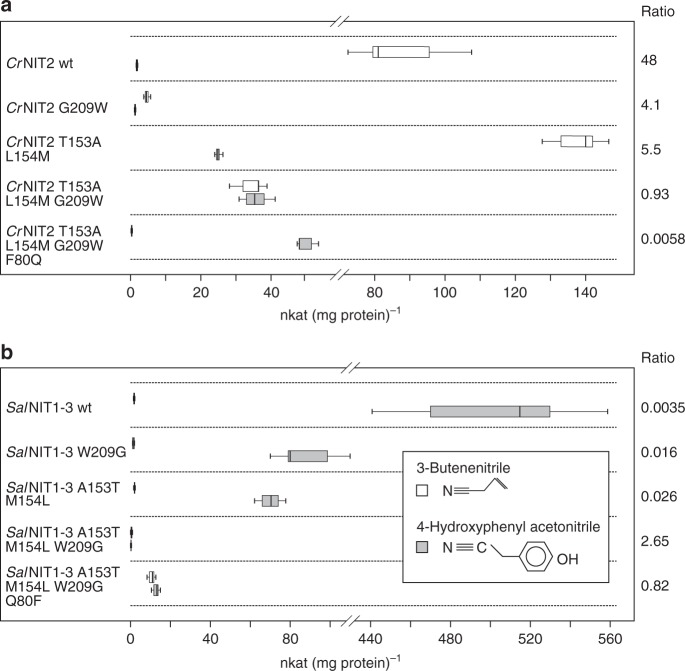
Residue exchanges in the proposed substrate-binding pocket of *Cr*NIT2 and *Sal*NIT1-3. **a** *Cr*NIT2 to *Sal*NIT1-3 exchanges. **b** *Sal*NIT1-3 to *Cr*NIT2 exchanges. Activities are given in nkat (mg protein)^−1^ (1st quartile, median and 2nd quartile ± sd)

By exchanging both motifs in *Cr*NIT2 (*Cr*NIT2 T153A L154M G209W), we could increase its activity with 4-hydroxyphenylacetonitrile by nearly 20-fold while the activity with 3-butenenitrile dropped to ~40% (Fig. 7a). Thus, a reasonable change in substrate specificity was obtained with only a minor decrease in catalytic efficiency. Additional mutation of the twist-determining residue (F80Q) however, had an adverse effect on catalytic efficiency, but further enhanced specificity for 4-hydroxyphenylacetonitrile. The complementary experiment with *Sal*NIT1-3 was similarly effective (activity against 4-hydroxyphenylacetonitrile dropped 39-fold, while activity against 3-butenenitrile increased 6-fold) but this required the exchange of the twist-determining residue Q80F and activity in total was strongly reduced (Fig. 7b). In summary, exchanging the residues in the active site between *Cr*NIT2 and *Sal*NIT1-3 had the general effect of decreasing activity for the wild-type substrate and increasing activity against the preferred substrate of the other enzyme.

## Discussion

Nitrilases constitute a system of enzymes with a wide range of specificities. This feature makes them attractive biocatalysts and nitrilases with specific properties are often sought by genomic mining or directed evolution^12,17,34^. Our experiments show that nitrilases regulate substrate specificity in two ways, both by the usual means of changing active-site residues, but also by altering quaternary structure induced by mutating interfacial residues. The implications of this are profound and should fundamentally change our understanding of the evolution of this enzyme class.

The starting point of this study was the observation that two nitrilases from the plant *Capsella rubella* (*Cr*NIT1 and *Cr*NIT2), although being virtually identical in the proposed substrate-binding pocket (Fig. 1) displayed clearly different substrate preferences (Fig. 2). These two enzymes belong to the NIT1 group of plant nitrilases, that are only found in plants of the Brassicaceae family and their proposed function is the catabolism of nitriles, which are derived from glucosinolates, typical secondary metabolites of this family^4,26,27^. The NIT2 enzymes are a subgroup within the NIT1 group which have been found in some members of the tribe *Camelineae* (*A. thaliana*, *A. halleri*, *A. lyrata*, *C. rubella*) and they have repeatedly evolved to change their substrate preference for short- vs. long-chained alkenenitriles, the reason for this possibly being the presence or absence of the short-chain glucosinolate sinigrin in the respective plant (our unpublished data).

By producing chimeras and using site-directed mutagenesis we identified a single residue, which is responsible for the preference of the enzyme for long-chained (H80 in *Cr*NIT1) or short-chained (F80 in *Cr*NIT2) nitriles. The default residue in this position in the plant NIT1 group is a histidine, in accordance with the general preference of these enzymes for long-chained nitriles^26^ but interestingly, it is also a phenylalanine in the NIT2 ortholog from *A. lyrata*, which also prefers the short-chain nitrile 3-butenenitrile. Moreover, the two NIT1 homologs *Sal*NIT1-3 and *Sar*NIT1-3 from *S. alba* and *S. arvensis*, respectively, which prefer arylnitriles as substrates, have different residues in this position (glutamine and tyrosine, respectively), while in the plant NIT4 homologs (usually highly specific for the substrate β-cyanoalanine) this position is occupied by a conserved arginine (Supplementary Figure 2). Interestingly this is correlated with a helical twist of Δφ = −74° ± 0.8° in all the NIT4 homologs reconstructed by us (Supplementary Figure 4a-c) (*At*NIT4 NP_197622.1 EMD-3497; *Lj*NIT4A BI420799 EMD-3504; *Ce*NIT NP_497791.1 EMD-3498). This evidence strongly suggests that this position, although presumably not located in the substrate-binding pocket, is involved in defining the substrate specificity of these enzymes.

Molecular modeling of the *Capsella* nitrilases clearly shows that the position is situated at the so-called C-interface, which is the interface connecting one nitrilase dimer to the next in the helix, and thus may exert an influence on the architecture of the helix. Indeed significant (Student’s two-tailed unpaired *t*-test: *p* = 0.036 and *p* = 0.039) changes in the twist of the helices are observed in the wild type and mutant enzymes which correlate with the substrate profile: enzymes which form helices of larger twist (*Cr*NIT2 and *Cr*NIT1 H80F) prefer the short-chain substrate and vice versa (Fig. 3a–d and Supplementary Movie 1). This principle seems to be of general validity, since it can be extended to other plant and bacterial nitrilases for which helical twist and substrate preference are known (Fig. 4). Thus it seems reasonable to assume that helical twist is a determiner of (or at least an indicator for) the substrate-binding-site architecture.

Remarkably, we were able to change the substrate specificity of one nitrilase (*Cr*NIT1 F80H or *Cr*NIT2) by mixing it with an active-site mutant of another enzyme of different twist (*Cr*NIT1 C182A or *Cr*NIT2 F80H C182A, respectively) (Fig. 5). This experiment clearly demonstrates that this change in substrate specificity can only be due to changes of the three-dimensional structure of the enzyme in the course of the experiment. Our explanation is that the active enzyme molecules become incorporated in helices of the active-site mutant (that is present in excess) and are imprinted with their twist. This experiment also potentially reveals a new way to engineer nitrilases with altered substrate specificities for biotechnological applications: the architecture of the binding pocket can be altered without changing its actual composition by mixing a candidate nitrilase with another nitrilase with a different twist. Such experiments may be hindered however, by the failure of the mixed enzymes to form heterocomplexes. We have observed earlier that the NIT4 homologs of *Sorghum bicolor* differ in their ability to form homo- or heterocomplexes^16^. Also, with other combinations of *Capsella* nitrilases (e.g., *Cr*NIT1 mixed with an excess of *Cr*NIT2 C182A) we did not see changes in substrate specificity, which, we think, is because the preformed homomeric nitrilase helices will not always readily exchange subunits with the candidate enzyme. This is supported by our observation that the *Cr*NIT2/*Cr*NIT2 F80H C182A mixture needed to be pre-incubated for 2 h at 37 °C to see an effect on substrate specificity.

**Fig. 8:**
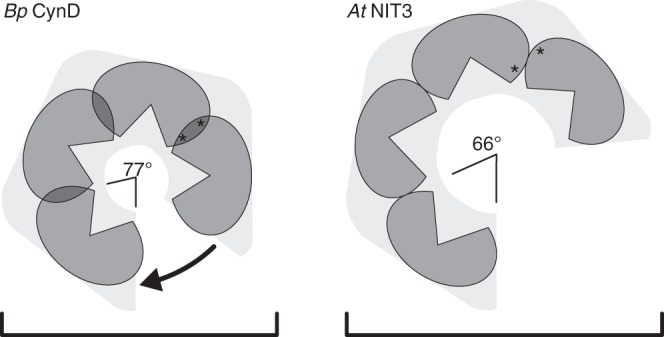
Schematic model of the nitrilase helix, viewed from above showing four dimers and comparing the geometry of *Bp* CynD (high specificity for cyanide) and *At*NIT3 (with a preference for large substrates). The position of the active sites, lying on either side of the two-fold symmetric C-interface is shown (*). *Bp* CynD has a smaller diameter and each subunit is more compressed at the interface between adjacent subunits (C-interface)

How can the influence of the helical twist on substrate specificity be explained? In the crystallized members of the C–N hydrolase superfamily, the outer border of the substrate-binding pocket is bounded by, in some cases a loop, and in others a short helix (Fig. 6, turquoise region). There are two close homologs to the helical nitrilases for which atomic resolution structural information is available which show helical associations: the β-alanine synthase from *Drosophila melanogaster* (pdb id: 2vhh)^35^ and Nit6803 from *Synechocystis* sp. PCC6803 where the helix or loop is held in place by interacting with its symmetry-related counterpart across the C-interface. In the case of β-alanine synthase this loop is disordered at either end of the octameric spiral of monomers and cannot be seen in the crystal structure suggesting that the interactions at the C-interface are responsible for maintaining the integrity of the active site. In these crystal structures there are two-fold symmetric interactions of one of two loops that are not present in the structures of other C–N hydrolase superfamily members that do not have spiral quaternary structures. Our experiments suggest that residue 80 plays a key role in this interface in *Cr*NIT1 and *Cr*NIT2 and that the details of this interaction play a role in defining the size of the active site cavity (Fig. 6). Correlated with this change in size is a change in the helical twist of the oligomeric spiral, making it tempting to suggest that tightening the spiral constricts the active site (Fig. 8 and Supplementary Movie 1). Such a change would have a strong impact on large substrates, while small substrates would still be able to enter the active site, which is in accordance with our results showing that *Cr*NIT1 and *Cr*NIT1 H80F have comparable activities with 3-butenenitrile (Fig. 2).

The influence of the quaternary structure on the architecture of the substrate-binding pocket in nitrilases has been unnoticed so far and may explain why efforts to change their substrate specificity by a directed exchange of residues within the proposed substrate-binding pocket have been largely unsuccessful until now. Thus, we attempted to change the substrate specificity in two nitrilases of similar twist but significantly different substrates (*Cr*NIT2 preferring 3-butenenitrile and *Sal*NIT1-3 preferring 4-hydroxyphenylacetonitrile) by exchanging residues of the binding pocket (Fig. 6). In both cases, substrate specificities could be changed substantially (Fig. 7) presumably without altering the helical twist. The final *Cr*NIT2 mutant (*Cr*NIT2 T153A L154M G209W) had the same activity with 3-butenenitrile and 4-hydroxyphenylacetonitrile and retained relatively high activity. The final *Sal*NIT1-3 mutant (*Sal*NIT1-3 Q80F A153A M154L W209G) was even more effective regarding the change in substrate specificity, however, at the expense of catalytic efficiency. This is, to our knowledge, the first time that substrate specificity of a nitrilase was substantially changed in a predictable manner.

With these results in mind, a possible course of enzyme evolution for the Brassicaceae NIT1 homologs may be delineated as follows: After a gene duplication (likely during the last whole genome duplication in the Brassicaceae family), the prototype NIT1 arose by mutations which changed the protein interface, resulting in an enzyme complex with smaller twist and thus a larger substrate-binding pocket. In this way the ubiquitous and highly specific predecessor NIT4 (large absolute twist, presumably small substrate-binding pocket) turned into an enzyme, which was less specific and was able to catabolize medium- to long-chain aliphatic nitriles. Such nitriles are breakdown-products of methionine-derived, chain-elongated glucosinolates, which are specific for the Brassicaceae family. Later, further gene duplications and mutations in certain Brassicaceae species resulted in the formation of additional NIT1 homologs, which, by further alteration of the twist (larger twist, smaller substrate binding-pocket) and possibly also changing the amino acid residues within the substrate binding-pocket, became specific for certain specialized nitriles of these species.

## Methods

### Homology modeling

The target protein sequences were aligned using the GenTHREADER^36^ and Fugue^37^ web servers to Nit6803 (pdb id: 3wuy)^21^, currently the only available spiral/helical nitrilase (EC. 3.5.5.1) structure available. Homology models were built with MODELLER^38^ within UCSF Chimera^39^. No attempt was made to model the N- and C-terminal regions that extended beyond the template.

### Overlap extension PCR and site-directed mutagenesis

Overlap extension PCR was performed using KAPA HiFi DNA polymerase (PEQLAB Biotechnology) to construct chimeric enzymes according to the method of Wurch et al.^40^. Site-directed mutagenesis based on the QuikChange^TM^ protocol was performed with partially overlapping primers^41^ also using KAPA HiFi DNA polymerase. Primers were designed by attempting to fulfill the following criteria: a minimum of 8 non-overlapping 3′ bases; G/C at both ends; *T*_m_ > 64 °C; mutation > 4 bases from 5′ end and 6–8 bases from the 3′ end. Successful plasmid construction and mutagenesis was confirmed by DNA sequencing.

### Expression and purification of nitrilases

Expression and purification of nitrilases was performed as described earlier^24,25,27^: Coding sequences of plant nitrilases were cloned into pET-21b(+) (Novagen) and expressed as C-terminal (His)_6_-tagged proteins in *Escherichia coli* BL21-CodonPlus (DE3)-RIL (Agilent Technologies). Bacteria were inoculated 1:20 from a pre-culture and grown overnight at 30 °C or 37 °C with constant agitation (220 rpm) in 600 mL 2YT broth containing ampicillin (100 μg mL^−1^) and chloramphenicol (30 μg mL^−1^) with the addition of 0–0.3 mM IPTG. The cells were pelleted at 4 °C and stored at −80 °C. The frozen cells were resuspended in 60 mL lysis buffer (50 mM sodium phosphate, 300 mM NaCl, pH 8.0) with 5 mM β-mercaptoethanol and 1 mg mL^−1^ lysozyme and incubated in a water/ice mixture in a bath sonicator (Sonorex RK 510, S. Bandelin) for 15 min. The cells were disrupted by sonication (B-17 sonifier, Branson) using a tapered micro tip in 30 s intervals for a total of 4 min and clarified by centrifugation at 13,000 × *g* for 30 min. The target protein was precipitated from solution by the gradual addition of ammonium sulfate to a final saturation of 40% with stirring on ice for 30 min. The protein was pelleted at 13,000 × *g* for 20 min, resuspended in 12 mL of lysis buffer and centrifuged (5000 × *g*, 5 min). The supernatant was loaded onto an equilibrated (10 column volumes of lysis buffer containing 10 mM imidazol) 2 mL Ni^2+^-NTA-agarose (Qiagen) column and washed with 5 column volumes of wash buffer (lysis buffer containing 40 mM imidazol). The protein was eluted with 1.25 column volumes of lysis buffer containing 250 mM imidazol. The eluent (2.5 mL) was loaded onto a PD-10 size exclusion column (GE Healthcare) and eluted with 3.5 mL of storage buffer (50 mM potassium phosphate, 0.1 mM DTT, pH 8.0). Aliquots of 500 μL were flash frozen in liquid nitrogen and stored at −80 °C. Protein samples destined for electron microscopy were additionally separated by gel-filtration on a Sephacryl S-300 HR column (GE Healthcare). The protein eluted as a range of sizes, the leading edge of the high molecular weight fraction was selected.

### Activity assays

Activity measurements were conducted as described^16^ in triplicate using substrates purchased from Lancaster Synthesis or Sigma-Aldrich. In the case of mixing experiments, single enzymes and enzyme mixtures were pre-incubated for 2 h at room temperature or 37 °C before assaying. The reaction tubes contained enzyme (0.1-10 μg), 50 mM potassium phosphate pH 8.0, 1 mM DTT and 2.5 mM substrate in a total volume of 1 mL. Negative controls, consisting of heat-denatured enzyme (incubated at 100 °C for 10 min), were tested in parallel to every experimental condition. Samples were incubated at 37 °C and a time-series of four samples were taken in the linear range at intervals of between 5 and 30 min. The nitrilase reaction was halted by pipetting 100 μL of sample into pre-prepared glass tubes (10 mL) containing 100 µL sodium phenolate (0.33 M) at every time point. Each 100 µL of sodium hypochlorite (20 mM) and sodium pentacyanonitrosylferrate (II) (0.01% [w/v]) were immediately added to the glass tubes, which were placed in boiling water for 2 min. After cooling to room temperature, 600 μL of distilled water was added to the solution and the extinction at 640 nm was recorded. A standard concentration curve was constructed with NH_4_Cl standards and used to determine the ammonia concentration in each sample. After ensuring that the reaction was still in the linear range, the specific activity was calculated for each enzyme/substrate combination.

### Negative-stain electron microscopy

Nitrilase filaments were negatively stained according to standard practices (e.g., Booth et al.)^42^. The purified protein solution (2.5 μL) was pipetted onto glow discharged carbon-coated 3 mm copper grids. The protein was allowed to adhere for 30 s, blotted, washed three times with distilled water and stained with uranyl acetate, blotted until a thin film was achieved and allowed to dry at room temperature. Samples were loaded into a FEI/Tecnai F20 FEGTEM equipped with a 4k × 4k CCD camera (GATAN US4000 Ultrascan, California, USA) and imaged at a sampling of 2.11 Å per pixel at 200 kV with a defocus of 300–500 nm under standard low-dose conditions.

### Helical image processing

Vertically aligned helical segments with 90% overlap were interpolated down by a factor of 2, normalized and reconstructed using the iterative helical real-space reconstruction (IHRSR)^43^ algorithm using SPIDER^44^. The helical twist and rise were estimated from the diffractogram calculated from vertically orientated helical segments and refined during the IHRSR procedure. Failure to converge on the correct helical twist results in a failure of the electron density map to converge on a stable solution. Convergence, measured by no further change in helical symmetry, occurred after approximately 30 iterations of the IHRSR algorithm. Twist-errors were estimated by dividing the data into thirds and producing three independent reconstructions. The Student’s two-tailed unpaired *t*-test was used to test for significance.

### Data visualization

All molecular visualization and high-quality image rendering was performed using UCSF Chimera^39^. Threshold values were calculated from the predicted volume enclosing the molecular weight of the protein complex using an average protein density value^45^ of 0.73 Da Å^−3^.

## Electronic supplementary material

Supplementary Information

Description of Supplementary Movie

Supplementary Movie 1

## Data Availability

All relevant data are available from the authors. Data from the helical reconstructions have been deposited in the Electron Microscopy Data Bank (EMDB, http://www.ebi.ac.uk/pdbe/emdb/): *At*NIT1 EMD-3486; *At*NIT3 EMD-3496; *At*NIT4 EMD 3497; *Ce*NIT EMD-3498; *Cr*NIT1 EMD-3499; *Cr*NIT1 H80F EMD-3500; *Cr*NIT2 EMD-3501; *Cr*NIT2 F80H EMD-3503; *Lj*NIT4A EMD-3504; *Sal*NIT1-3 EMD-3505. Protein and DNA sequences are available from Genbank with the following accession numbers: *At*NIT1 NP_851011.1; *At*NIT3 NP_190018.1; *At*NIT4 NP_197622.1; *Cr*NIT1 XP_006291436.1; *Cr*NIT2 XP_006284056.1; *Ce*NIT NP_497791.1; BpCynD AAN77004.1; *Lj*NIT4A BI420799.1; *Sal*NIT1-3 MH567030.
