# Supplementary material for: Substrate specificity of plant nitrilase complexes is affected by their helical twist
Source: Commun Biol. 2018 Nov 2;1:186. doi: 10.1038/s42003-018-0186-4 (PMC6214922; doi:10.1038/s42003-018-0186-4)
Supplement: Supplementary file 1 — Supplementary Information [file 42003_2018_186_MOESM1_ESM.pdf]

```
CrNIT1       1 MSSTVKNTADPVDGVAPSSIVRVTIVQASTVYNNTP-------ATLDKAEKYVVEAASKG
CrNIT2       1 MSGTEQIMSTKFNGVASPTTVRATIVQASTVYNDTP-------ATLEKAKKYIEEAASNG
SalNIT1-3    1 MSKTTLKDTTPVSNDFPSTIVRATIVQASSVYNDTP-------KTLEKAEKFIAEAASDG
Nit6803      1 MLGK--IMLN------YTKNIRAAAAQISPVLFSQQ-------GTMEKVLDAIANAAKKG
DmβAS       53 FDIKGYRFTAREEQTRKRRIVRVGAIQNSIVIPTTAPIEKQREAIWNKVKTMIKAAAEAG

CrNIT1      54 AKLVLFPEAFVGGYPRGFRFGLATGVHNEEGRDEFRKYHASAIKVPGPEVERLAELAGKT
CrNIT2      54 SELVVFPEAFIGGYPRGFRFGLAVGVFNEEGRDEFRKYHASAIPVPGPEVEKLADMAGKN
SalNIT1-3   54 AQLVVFPEAYVGGYPRGFRFGMAVGVQNEEGRDLFRKYHASAIAVPGPEVDKLAEMARKY
Nit6803     46 VELIVFPETFVPYYPYFSFVEPPVLM-----GKSHLKLYQEAVTVPGKVTQAIAQAAKTH
DmβAS2     113 CNIVCTQEAWT------MPFAFCTR-----EKFPWCEFAEEA--ENGPTTKMLAELAKAY

CrNIT1     114 NVYLVMGAIEKD---GYTLYCTALFFSPQGQFLGKHRK-LMPTT---LERCIWGQGDGST
CrNIT2     114 NVYLVMGAIEKD---GYTLYCTALFFSPQGQFLGKHRK-LMPTT---LERCIWGQGDGST
SalNIT1-3  114 KVYLVTGAIEKD---GYTLYCIALFFSSEGHFLGKHRK-VMPTA---MERVIWGYGDGST
Nit6803    101 GMVVVLGVNERE---EGSLYNTQLIFDADGALVLKRRK-ITPTY---HERMVWGQGDGAG
DmβAS      160 NMVIIHSILERDMEHGETIWNTAVVISNSGRYLGKHRKNHIPRVGDFNESTYYMEGN-TG

CrNIT1     167 IPVYDTPIGKLGAAICWENRMPLYRTALYAKGIEIYCAPT------AD----GSKEWQSS
CrNIT2     167 IPVYDTPIGKLGAAICWENRMPLYRTALYAKGIEIYCAPT------AD----GSKEWQSS
SalNIT1-3  167 LPVYDTPLGKLGGAICWENRMPSLRTSLYAKGVELYCAPT------AD----WSKEWQSS
Nit6803    154 LRTVDTTVGRLGALACWEHYNPLARYALMAQHEQIHCGQFPGSMVGQI----FADQMEVT
DmβAS      219 HPVFETEFGKLAVNICYGRHHPQNWMMFGLNGAEIVFNPS------ATIGRLSEPLWSIE

CrNIT1     217 MMHIALEGGCFVLSACQFCLRKDFPDHPDYLFTDWEDYKEDDA-IVSQGG--SVIISPLG
CrNIT2     217 MLHIAIEGGCFVLSACQFCQRKDFPEHPDYLFTDLDENKEQDA-IVSQGG--SVIISPLG
SalNIT1-3  217 VMHIAMEGGCFVMAACQFCLRKDYPEDPNYYFTDESDDHSPEA-VVSPGG--SVIISPLG
Nit6803    210 MRHHALESGCFVINA----TGWLTAEQKLQITT---DEKMHQA-L--SGGCYAIISPEG
DmβAS      273 ARNAAIANSYFTVPINRV-GTEQFPNEY----TSGDGNKAHKEFGPFYGS--SYVAAPDG

CrNIT1     274 QVLAGPNFESEGLITADLDLGDVARAKLYFDAVGHYSRPDVLHLTVNEHPK---------
CrNIT2     274 QVLAGPNFESEGLITADLDLGEIARAKLYFDVVGHYTKPDVLSLTVNEDPK---------
SalNIT1-3  274 KILAGPNFGSEGLVTADLDLGDIAKAKLYFDVVGHYSRPDIFNLTVNEKEK---------
Nit6803    260 KHLCEPIAEGEGLAIADLDFSLIAKRKRMMDSVGHYARPDLLQLTLNNQPWSALEANPVT
DmβAS      326 SRTPSLSRDKDGLLVVELDLNLCRQVKDFW---GFRMTQRVPLYAESFKKA---------

CrNIT1     325 -KTVTFMTKVEKAEDDSNK--------------
CrNIT2     325 -KTVTFVSKVEKAEDASNK--------------
SalNIT1-3  325 -KPVTFVSKPEDDSEPQDK--------------
Nit6803    320 PNAIPAVSDPELTETIEALPNNPIFSH------
DmβAS      374 -SEHGFKPQIIKETQFPGDDDDKHHHHHHHHSG
```

Supplementary Figure 1 Sequence alignment of C-N hydrolase family members relevant for this work. Sequence conservation is indicated by black (100% identity) or grey (100% similarity) boxes. Identical residues in *Cr*NIT1 and *Cr*NIT2 are shown in blue boxes. The residues forming the catalytic tetrad are indicated with magenta boxes. H80/F80 of the *Capsella* nitrilases are boxed turquois. The extended N-terminal region of *Dm*βAS was removed from the alignment. The ClustalW alignment was manually modified based on pairwise structural alignments to Nit6803 and *Dm*βAS by FUGUE. *Cr*NIT1, *Cr*NIT1: nitrilase 1 and 2, respectively, from *Capsella rubella*, *Dm*βAS: β-alanine synthase from *Drosophila melanogaster*, Nit6803: nitrilase from *Synechocystis* sp. PCC6803, *Sal*NIT1-3: nitrilase 1-3 from *Sinapis alba*

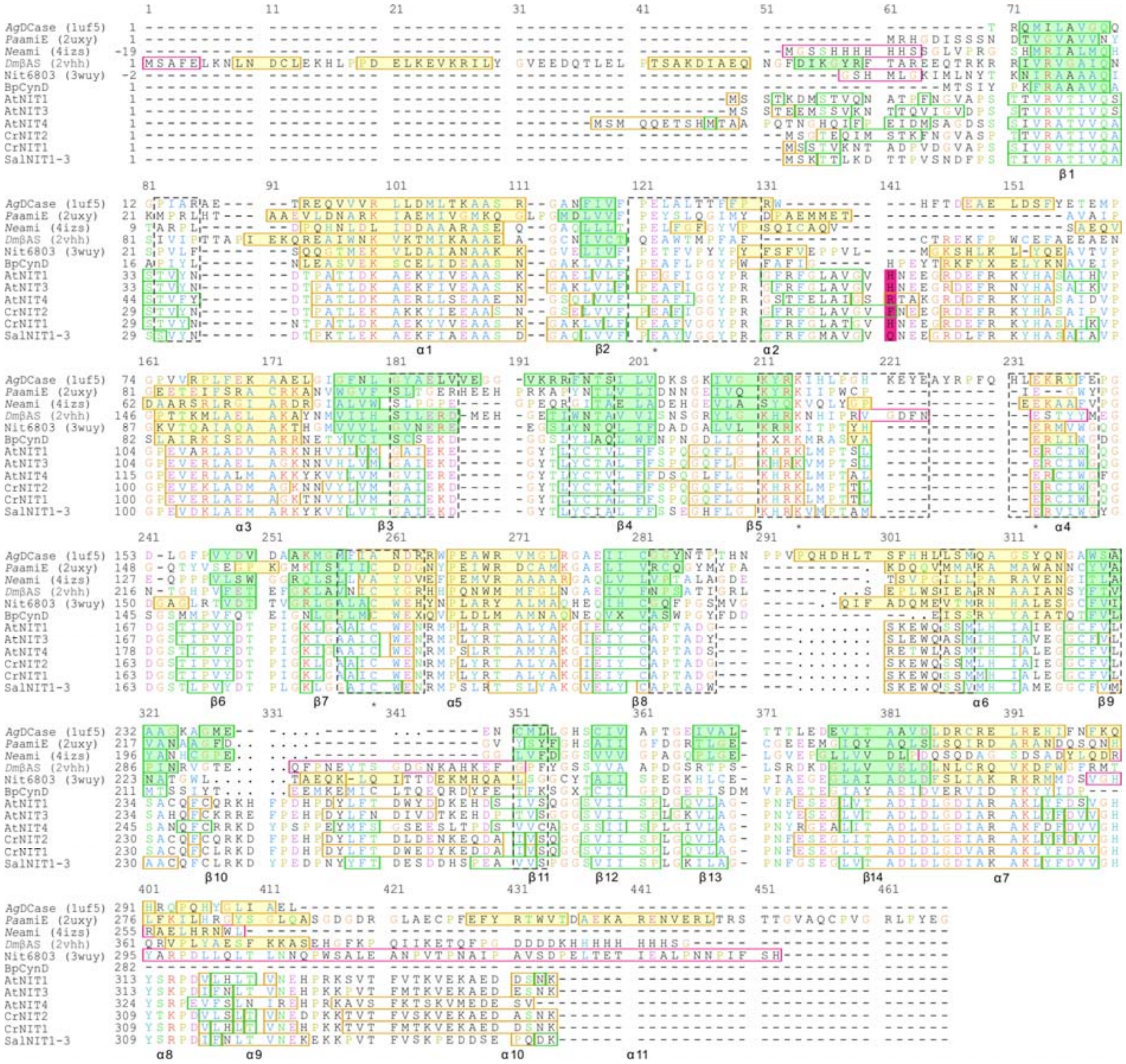

Supplementary Figure 2 Sequence alignment from structural superposition[1] of crystallized C-N hydrolase family members relevant for this work (1uf5, 2uxy, 4izs, 2vhh, 3wuy) and proteins for which crystal structures are unavailable (*Bp*CynD, *At*NIT1, *At*NIT3, *At*NIT4, *Cr*NIT1, *Cr*NIT2, *Sal*NIT1-3) aligned using BLOSUM62. Secondary structure is shown in yellow- (helices) or green (strands) boxes, GOR[2] secondary structure predictions are shown as outlines, helices and strands have been numbered according to Thuku *et al*. (2009)[3]. Magenta outlines indicate regions not present in the crystal structures. Residues forming the catalytic tetrad are indicated with stars. H80/F80 of the *Capsella* nitrilases are boxed in magenta along with the corresponding residues from the other relevant plant nitrilases. Regions within 10 nm of the active-site cysteine in any nitrilase superfamily structure are shown with a dashed outline (see Supplementary Fig. 3). *Ag*DCase (1uf5): C171A/V236A mutant of *N*-carbamyl-D-amino acid amidohydrolase from *Agrobacterium* sp. KNK712 complexed with *N*-carbamyl-D-methionine, *Paami*E (2uxy): amidase from *Pseudomonas aeruginosa* with trapped acyl transfer intermediate, *N*eami (4izs): C145A mutant of the amidase from *Nesterenkonia* AN1 complexed with butyramide substrate; *Dm*ßAS (2vhh): β-alanine synthase from *Drosophila melanogaster*; Nit6803 (3wuy): nitrilase from *Synechocystis* sp. PCC6803; *Bp*CynD: cyanide dihydratase from *Bacillus pumilus*; *At*NIT1, *At*NIT3, *At*NIT4: nitrilase 1, 3 and 4 from *Arabidopsis thaliana*; *Cr*NIT1, *Cr*NIT2: nitrilase 1 and 2, from *Capsella rubella*; *Sal*NIT1-3: nitrilase 1-3 from *Sinapis alba*

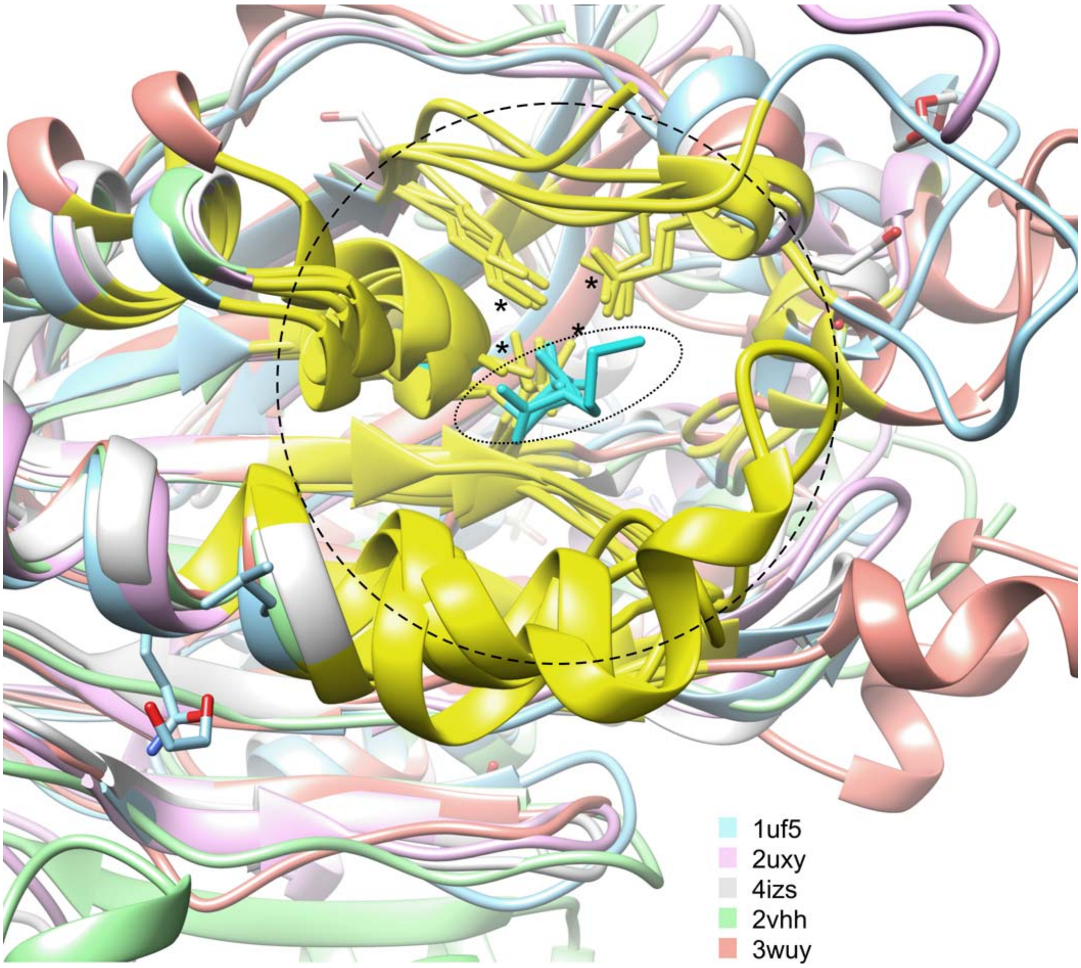

Supplementary Figure 3 Structural superposition[1] of crystallized C-N hydrolase family members relevant for this work (1uf5, 2uxy, 4izs, 2vhh, 3wuy). Residues forming the catalytic tetrad are indicated with stars. Regions within 10 nm of the active-site cysteine in any nitrilase superfamily structure are shown with a dashed outline and colored yellow (see Supplementary Fig. 2). This region extends beyond the catalytic tetrad as well as those amino acids interacting with co-crystallized substrates and substrate intermediates (colored cyan with finely dashed outline) in any of the crystallized nitrilase homologues. The structurally conserved nitrilase superfamily core can be seen. 1uf5: C171A/V236A mutant of *N*-carbamyl-D-amino acid amidohydrolase from *Agrobacterium* sp. KNK712 complexed with *N*-carbamyl-D-methionine, 2uxy: amidase from *Pseudomonas aeruginosa* with trapped acyl transfer intermediate, 4izs: C145A mutant of the amidase from *Nesterenkonia* AN1 complexed with butyramide substrate; 2vhh: β-alanine synthase from *Drosophila melanogaster*; 3wuy: nitrilase from *Synechocystis* sp. PCC6803

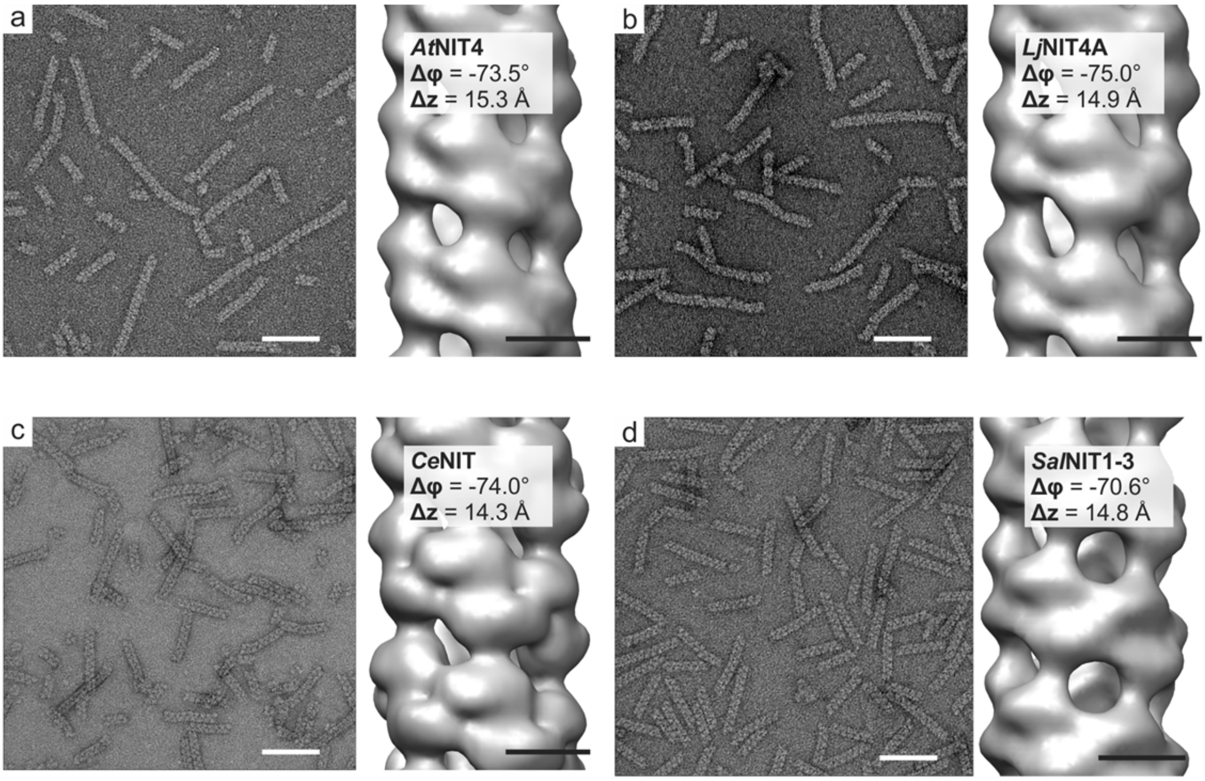

Supplementary Figure 4 Reconstruction of additional nitrilase helical structures from negative-stained electron-micrographs. **a** *Arabidopsis thaliana* NIT4, **b** *Lotus japonicus* NIT4A, **c** *Caenorhabditis elegans* NIT, **d** *Sinapis alba* NIT1-3. Scale bars represent 50 nm on the electron micrographs, 5 nm on the reconstructed fibers

Supplementary Table 1 Substrate profiles of *At*NIT1 and *At*NIT3 from *A. thaliana* and *Cr*NIT1-*Cr*NIT3 from *C. rubella* measured with aliphatic nitriles of different length. The data show means ± standard deviations (n = 4-9). *n.d.: not detectable

	Specific enzyme activity [nkat (mg Protein)$^{-1}$]				
	A. thaliana		*C. rubella*		
Substrate	*At*NIT1	*At*NIT3	*Cr*NIT1	*Cr*NIT2	*Cr*NIT3
Acetonitrile	0.51±0,58	0.59±0.0.72	n.d.	0.367±0.58	n.d.
Propionitrile	n.d.*	n.d.	n.d.	n.d.	n. d.
Butyronitrile	9.45±1.56	0.70±1.57	8.02±1.89	1.17±1.32	0.44±0.73
Pentanenitrile	167.07±13.50	27.64±13.59	136.36±24.30	3.51±1.64	6.44±2.51
Heptaneitrile	304.44±72.58	53.53±15.18	304.07±74.23	1.97±2.99	11.11±2.49
Octanenitrile	353.49±97.79	67.07±16.68	268.11±40.91	0.88±1.05	22.54±6.38
Nonanenitrile	141.33±27.61	82.06±11.93	193.38±45.49	0.33±0.80	32.99±9.07
Dodecanenitrile	9.42±7.67	37.19±15.32	25.24±12.24	n.d.	11.99±3.60

Supplementary references

1. Meng E. C., Pettersen E. F., Couch G. S., Huang C. C. & Ferrin T.E. Tools for integrated sequence-structure analysis with UCSF Chimera. *BMC Bioinformatics*. **7**, 339 (2006).
2. Gibrat J. F., Garnier J. & Robson, B. Further developments of protein secondary structure prediction using information theory. New parameters and consideration of residue pairs. *J. Mol. Biol.* **198**, 425-443 (1987).
3. Thuku, R. N., Brady, D., Benedik, M. J. & Sewell, B.T. Microbial nitrilases: versatile, spiral forming, industrial enzymes. *J. Appl. Microbiol.* **106**, 703-727 (2009).
