# Supplementary material for: Substrate specificity of plant nitrilase complexes is affected by their helical twist
Source: Commun Biol. 2018 Nov 2;1:186. doi: 10.1038/s42003-018-0186-4 (PMC6214922; doi:10.1038/s42003-018-0186-4)
Supplement: Supplementary file 2 — Description of Supplementary Movie [file 42003_2018_186_MOESM2_ESM.docx]

**Description of Additional Supplementary Files**

**File Name: Supplementary Movie 1**

**Description:** Animated gif demonstrating structural changes between *Cr*NIT1 and *Cr*NIT2 helices. A ~1.5° change in helical twist leads to large-scale conformational changes at the supramolecular interfaces. A circle indicates the approximate position of one of the proposed active-site pockets, which lie on either side of the two-fold symmetric C-interface. The helical twists of *Cr*NIT1 and *Cr*NIT2 are indicated, along with their preferred substrates. As the absolute value of the helical twist increases, we observe a spring tightening effect. Individual monomers can be seen to move across the A-interface leading to undetermined rearrangements at the binding pocket. This visualization was created by imposing five equally spaced helical twists between -69.7° and -68.5° on *Cr*NIT images over 30 cycles of IHRSR refinement. The resulting reconstructions, representing a transition between the two helical states, are presented here in succession.
