# Supplementary figures and images for: Substrate specificity of plant nitrilase complexes is affected by their helical twist
Source: Commun Biol. 2018 Nov 2;1:186. doi: 10.1038/s42003-018-0186-4 (PMC6214922; doi:10.1038/s42003-018-0186-4)

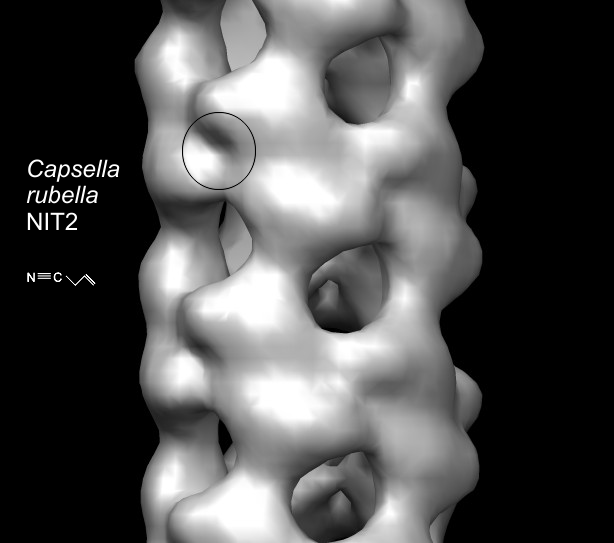

Supplement: Supplementary file 3 — Supplementary Movie 1 [file 42003_2018_186_MOESM3_ESM.gif]
